# Integrating Chronophysiological and Bioinformatic Perspectives on Nesfatin-1, Circadian Feeding Rhythms, and Cortisol Regulation

**DOI:** 10.3390/ijms27219942

**Published:** 2026-11-06

**Authors:** Norsham Juliana, Izuddin Fahmy Abu, Sahar Azmani, Srijit Das, Nazefah Abdul Hamid, Amirah Azzeri, Mohd Hafiz Jaafar, Sofwatul Mokhtarah Maluin, Marjanu Hikmah Elias

**Affiliations:** 1 Faculty of Medicine and Health Sciences, Universiti Sains Islam Malaysia (USIM), Nilai 71800, Negeri Sembilan, Malaysia; njuliana@usim.edu.my (N.J.); drazmanisahar@usim.edu.my (S.A.); nazefah@usim.edu.my (N.A.H.); amirah.azzeri@usim.edu.my (A.A.); dr.hafizjaafar@usim.edu.my (M.H.J.); sofwatulmal@usim.edu.my (S.M.M.); marjanuhikmah@usim.edu.my (M.H.E.); 2 Institute of Medical Science Technology, Universiti Kuala Lumpur, Kajang 43000, Selangor, Malaysia; 3 College of Medicine and Health Sciences, Sultan Qaboos University, Muscat 123, Oman; s.das@squ.edu.om

**Keywords:** nesfatin-1, cortisol, circadian rhythm, hypothalamic–pituitary–adrenal axis, circadian misalignment, shift work, NUCB2, steroidogenesis

## Abstract

Nesfatin-1, a peptide derived from nucleobindin-2, regulates feeding, stress, and sleep–wake processes. To date, the available information suggests that nesfatin-1 is involved in stress regulation and colocalises with substances that influence cortisol, but the circadian interplay between nesfatin-1 and cortisol remains poorly defined. This review integrates biological timing, tissue-specific actions of the hypothalamic–pituitary–adrenal axis, and circadian misalignment, and is supported by in silico protein-association analysis. A structured database search retained relevant studies published from January 2006 to March 2026; heterogeneity necessitated narrative synthesis. Rodent experiments support effects of central NUCB2/nesfatin-1 on feeding and HPA-axis activation. Murine corticotroph experiments report increased POMC abundance, whereas an H295R human adrenocortical carcinoma-cell study reports reduced cortisol content and stimulated release. These findings concern different experimental systems and do not demonstrate coordinated central–adrenal regulation in vivo. The network analysis identified NPY and POMC as prominent nodes within the predefined candidate network, with enrichment of feeding- and HPA-axis-related pathways. Given the biologically targeted selection of input genes, these findings are interpreted as hypothesis-generating associations rather than independent confirmation of a regulatory mechanism. Within the literature examined in this review, direct evidence characterising the 24 h temporal profile of nesfatin-1 in humans, its phase relationship with cortisol, and potential alterations during circadian misalignment was not identified. This apparent evidence gap warrants further investigation through controlled, time-resolved human studies.

## 1. Introduction

Nesfatin-1 is an 82-amino-acid peptide derived from nucleobindin-2 (NUCB2) and was first identified as a leptin-independent satiety signal in the hypothalamus. It is expressed in several hypothalamic and brainstem regions involved in feeding, autonomic control, and stress responses, including the paraventricular nucleus (PVN), supraoptic nucleus (SON), arcuate nucleus, and nucleus tractus solitarius. In the PVN and SON, subsets of nesfatin-1 neurons co-express oxytocin, vasopressin, or corticotropin-releasing hormone (CRH), and refeeding activates these neuronal populations [1,2].

Importantly, NUCB2 mRNA expression, tissue NUCB2/nesfatin-1 immunoreactivity, circulating nesfatin-1 concentrations, and responses to exogenously administered nesfatin-1 represent distinct experimental measures and should not be interpreted interchangeably; the localisation of recognised antigens circulating measurements may not directly reflect central nesfatin-1 activity [3]. Likewise, responses to exogenously administered nesfatin-1 do not necessarily establish the physiological role of the endogenous peptide [4,5].

The available evidence also has a temporal dimension. In rats, PVN NUCB2 expression rises during the early light phase, when these nocturnal animals normally eat less, and interference with PVN NUCB2 increases light-phase food intake [6]. Separately, central nesfatin-1 activates CRH neurons and increases circulating adrenocorticotropic hormone (ACTH) and corticosterone in rodents, whereas experiments in human adrenal cortical cells indicate a direct inhibitory effect on cortisol synthesis [7,8]. These apparently different actions suggest that nesfatin-1’s effect on the stress axis may depend on the site of action and physiological context.

Despite these observations, evidence concerning nesfatin-1 in feeding rhythm, sleep, and hypothalamic–pituitary–adrenal (HPA) axis regulation have largely been examined separately. Although experimental studies have demonstrated central activation of HPA-axis pathways and inhibitory effects on cortisol production in human adrenal-derived cells, these findings originate from different biological models and experimental conditions. Their relevance to the temporal coordination of feeding and stress responses therefore remains uncertain. In particular, the 24 h profile of nesfatin-1 in humans and its temporal relationship with cortisol have not been adequately characterised. While existing reviews have described the broader physiology of nesfatin-1, including its roles in food intake, metabolism, and stress-related pathways [5,9,10], an integrated evaluation of its chronophysiological and tissue-specific actions remains limited.

The present review addresses these limitations by integrating three complementary perspectives: daily biological timing, tissue-specific actions within the HPA axis, and circadian misalignment. Its contribution lies in critically comparing findings across experimental models, particularly the contrasting effects of central nesfatin-1 administration on HPA-axis activation and direct peptide exposure on cortisol production in human adrenal-derived cells. It also distinguishes observations of daily or state-dependent variation from evidence of endogenous circadian rhythmicity, a distinction essential for interpreting the available chronophysiological findings. Complementary protein-association analysis is incorporated as an exploratory approach to prioritise candidate molecular associations for future investigation, without implying causality or independent validation of a physiological mechanism. Rather than establishing a novel circadian interaction, this review identifies three key translational gaps: the lack of comprehensive 24 h human nesfatin-1 profiles, direct temporal comparisons with cortisol, and studies examining these relationships during circadian misalignment. Collectively, these analyses provide a hypothesis-generating framework for future investigations into the potential role of nesfatin-1 as a marker, mediator, or compensatory response in metabolic and neuroendocrine adaptation to circadian disruption.

## 2. Evidence Synthesis and Molecular Network Analysis

### 2.1. Synthesis of Current Evidence

Eligible studies were retained for qualitative synthesis and organised according to the key domains of interest within the scope of this review. Table 1 summarises the available evidence, its strengths and limitations, and the domains in which important evidence gaps remain.

#### 2.1.1. Daily and State-Dependent Variation in NUCB2/Nesfatin-1

The included evidence on daily nesfatin-1 regulation comprised time-of-day comparisons of circulating concentrations under dietary or exercise conditions and an experimental study of sleep-dependent hypothalamic expression. These studies provided evidence of context-dependent variation but did not establish an endogenous circadian rhythm of nesfatin-1 secretion [11,12,13,22].

Evidence for daily variation is currently based mainly on rodents. In rats, PVN NUCB2 mRNA increases during the early light phase in parallel with reduced food intake. Immunoneutralization or PVN-selective knockdown increases light-phase feeding, whereas the normal rise is blunted in hyperphagic Zucker-fatty rats [4]. PVN-specific NUCB2 knockdown in mice similarly increases light-phase food intake and reduces plasma oxytocin [5]. In male mice, Mohan et al. (2014) measured circulating NUCB2/nesfatin-1 at the beginning and middle of the light phase and immediately before the dark phase [11]. Serum concentrations did not differ between dietary groups at the beginning of the light phase. However, the high-carbohydrate group had higher concentrations at the middle-light-phase measurement, whereas the high-fat, high-protein, and high-carbohydrate groups had lower concentrations than controls before the dark phase. These findings demonstrated time-specific dietary differences rather than a defined within-group daily waveform. The accompanying cell experiments showed nutrient-dependent regulation of NUCB2 expression and nesfatin-1 secretion, supporting nutritional state as an important consideration when interpreting circulating measurements [11].

Human exercise studies also identified differences across testing times. Algul et al. (2017) reported increased serum nesfatin-1 after night-time aerobic exercise, but no significant mean increase after morning exercise, in trained and untrained young men [12]. Morning testing followed overnight fasting, whereas evening testing followed a light meal, limiting attribution of the findings to clock time alone [12]. Ozcelik et al. (2018) similarly observed significant increases after morning and night soccer matches, but not afternoon matches; baseline concentrations did not differ significantly between sessions [13]. These results concern exercise-associated responses rather than spontaneous daily rhythmicity. Moreover, statistical significance within some sessions but not others does not itself demonstrate a difference in response between sessions [13].

Vas et al. (2013) provided adjacent evidence linking nesfatin-1 to sleep regulation. REM-sleep deprivation reduced hypothalamic NUCB2/nesfatin-associated transcript and protein measures, and rebound sleep restored these measures [22]. Central nesfatin-1 administration at lights-on also altered sleep–wake states. However, expression did not differ between undisturbed controls examined at lights-on and later in the early light phase. Thus, the study demonstrated sleep-state responsiveness without establishing a full-day expression rhythm [22].

#### 2.1.2. Nesfatin-1 and Feeding Rhythms

Central nesfatin-1 reduces food intake through pathways involving oxytocin, melanocortin signalling, and inhibition of orexigenic neuropeptide Y neurons [9,23]. Its administration during the rodent dark phase reduces meal size or prolongs intervals between meals, while PVN NUCB2 appears to restrain inappropriate feeding during the light phase [4,15]. Together, these studies link nesfatin-1 to the amount and timing of feeding in rodents. The feeding studies also showed suppression of nocturnal intake and changes in meal microstructure following central nesfatin-1 administration. Goebel et al. (2011) found that intracerebroventricular nesfatin-1 reduced dark-phase food intake in male mice through smaller meals, lower meal frequency, and longer inter-meal intervals [14]. The response had a delayed onset, whereas peripheral administration did not alter intake or feeding microstructure under the tested conditions. These findings support central effects on meal termination and meal spacing during nocturnal feeding, rather than demonstrating a shift in the daily phase of food intake [14].

Prinz et al. (2015) found that the active fragment nesfatin-1(30–59) affected feeding microstructure differently in normal-weight and diet-induced-obese rats [16]. Central administration reduced meal size in normal-weight rats, whereas obese rats showed reduced meal frequency and longer inter-meal intervals. Peripheral administration did not reduce intake. The findings indicate that metabolic or dietary conditions can influence the feeding response to the administered fragment, but do not establish whether endogenous full-length nesfatin-1 determines daily feeding rhythms [16].

Dong et al. (2014) provided complementary evidence from the dorsal vagal complex. Local nesfatin-1 administration reduced cumulative dark-phase intake, while repeated administration reduced body-weight gain [24]. Electrophysiological experiments showed altered activity of glucose-sensitive neurons in this region. However, the reported feeding outcomes concerned cumulative intake rather than redistribution of meals across light and dark phases [24]. Collectively, the feeding studies support effects on intake and meal organisation within the active phase, but do not demonstrate circadian entrainment or phase resetting of feeding [14,16].

#### 2.1.3. Nesfatin-1 and HPA-Axis Activity

The selected studies provided evidence of central HPA-axis activation and context-dependent systemic glucocorticoid responses. Yoshida et al. (2010) showed that restraint stress activated nesfatin-1-immunoreactive neurons in hypothalamic and brainstem regions without increasing circulating nesfatin-1 under the examined conditions [3]. Intracerebroventricular nesfatin-1 activated CRH-expressing paraventricular neurons and other stress-related neuronal populations, increased intracellular calcium in isolated CRH-immunoreactive neurons, and elevated plasma ACTH and corticosterone. These observations support a central link between nesfatin-1 signalling and HPA-axis output; the measured glucocorticoid was corticosterone, not cortisol [3].

Toru et al. (2015) reported a different pattern after chronic peripheral nesfatin-1 administration in rats [25]. Plasma cortisol was lower in nesfatin-1-treated animals subjected to intestinal ischemia/reperfusion than in animals exposed to ischemia/reperfusion alone, whereas nesfatin-1 increased cortisol in the laparotomy comparison. Thus, the direction of the systemic response depended on the experimental condition. Because the study measured circulating cortisol after systemic treatment, it did not identify whether the effect arose centrally, within the adrenal gland, or through other responses to injury. The authors also identified measurement of cortisol rather than corticosterone in rats as a limitation [25].

Nasri et al. (2024) supplied additional axis-related evidence through the mouse arm of their adrenal study: administration of NUCB1-derived nesfatin-1-like peptide altered adrenal steroidogenic and pituitary stress-related transcripts [10]. These gene-expression findings are distinct from demonstrated changes in circulating ACTH or glucocorticoid secretion and should not be interpreted as an additional independent study or as a nesfatin-1-specific in vivo hormonal response [10].

Hence, current evidence has not yet demonstrated a simple separation in which nesfatin-1 supports satiety during the biological night without engaging stress pathways. In rats, central nesfatin-1 suppresses feeding, but it can also activate the HPA axis and alter vigilance. One study found that rapid-eye-movement sleep deprivation reduced hypothalamic nesfatin-1/NUCB2 expression, whereas intracerebroventricular nesfatin-1 at light onset reduced rapid-eye-movement sleep and increased wakefulness and sleep fragmentation [22]. Thus, the relationships among satiety, stress, and sleep may depend on timing, dose, brain region, and physiological state.

#### 2.1.4. Direct Nesfatin-1–Cortisol Interaction

Direct adrenal evidence in the selected set came from Nasri et al. (2024), who examined nesfatin-1 and nesfatin-1-like peptide in human H295R adrenocortical carcinoma cells [10]. Both peptides reduced cellular cortisol content at the later incubation time tested, together with reductions in steroidogenic-enzyme transcripts. Peptide treatment also reduced CREB phosphorylation and attenuated forskolin-stimulated cortisol release. These findings support an inhibitory action on cortisol output in isolated adrenal cells and implicate signalling associated with the adenylyl cyclase/PKA/CREB pathway [10]. However, cortisol content and release are not direct measurements of steroid synthetic flux, and the signalling results do not establish every step of the proposed mechanism.

The isolated-cell findings differ from the central HPA activation reported by Yoshida et al. (2010), but the studies addressed different sites of action and different glucocorticoid outcomes [3]. They therefore do not establish a contradiction or determine the net physiological effect of endogenous nesfatin-1 [3]. The accompanying mouse experiments in Nasri et al. assessed systemic nesfatin-1-like peptide exposure and gene expression, rather than isolating a direct in vivo adrenal response. This evidence set did not resolve direct regulation of cortisol in primary human adrenal tissue or its contribution to whole-organism glucocorticoid regulation [10].

#### 2.1.5. Nesfatin-1 in Shift Workers

Shift work, particularly night work and irregular schedules, is associated with altered cortisol concentrations, rhythm, and cortisol awakening response, although findings vary according to sampling protocol and shift pattern [6]. No eligible study identified in this review directly characterised a 24 h nesfatin-1 rhythm in shift workers. Consequently, this set provided no direct evidence on circulating nesfatin-1 profiles, feeding rhythms, or nesfatin-1–cortisol relationships in shift workers. This is a limitation of the included evidence set, not a finding that relevant occupational research does not exist. The exercise timing and experimental sleep-deprivation studies should not be treated as substitutes for studies of occupational shift work, because their exposures and sampling designs addressed different questions [12,13,22].

Table 2 summarises the characteristics and key findings of available studies examining nesfatin-1 in five important domains described in Table 1. Collectively, these studies highlight the limited evidence addressing the temporal relationship between nesfatin-1 and HPA axis activity. Whether nesfatin-1 changes in phase, amplitude, or timing relative to cortisol during circadian misalignment therefore remains unknown.

### 2.2. Molecular Network and Functional Enrichment Analysis

#### 2.2.1. List of Genes Included in the In Silico Analysis

To construct a comprehensive network representing the intersection of nesfatin-1 signaling, chronophysiology, and glucocorticoid regulation, a multi-tiered seed expansion and literature curation workflow was conducted. Nucleobindin-2 (*NUCB2*, encoding nesfatin-1) served as the primary anchor seed gene. Protein–protein interaction (PPI) networks were retrieved from the STRING database (version [e.g., 12.0]; *Homo sapiens*, minimum interaction confidence score ≥ [e.g., 0.400 or 0.700]). First, primary (first-degree) physical and functional interactors directly associated with NUCB2 were queried (*n* = 15). To capture downstream and secondary regulatory pathways, each primary interactor was iteratively queried within STRING to harvest second-degree interaction partners (*n* = 140). In parallel, an empirical literature curation was conducted across PubMed/MEDLINE using Boolean combinations of terms encompassing circadian rhythms, feeding behaviors, and cortisol/hypothalamic–pituitary–adrenal (HPA) axis dynamics (e.g., “circadian feeding”, “cortisol regulation”, “HPA axis”, and “hypothalamic neuropeptides”), yielding *n* = 29 biologically validated candidate genes. Merging the STRING-expanded network (first- and second-degree interactors) with the literature-derived gene pool, followed by the systematic removal of duplicate entries and unverified isoforms, yielded a curated target cohort of exactly 100 non-redundant, unique genes (Table 3). All gene symbols were standardized according to current HUGO Gene Nomenclature Committee (HGNC) guidelines prior to downstream topological, functional enrichment, and pathway analyses.

#### 2.2.2. Topological Centrality Analysis

Across the centrality rankings, neuropeptide Y (NPY) and proopiomelanocortin (POMC) were the only proteins appearing in the top sets for betweenness, bottleneck, degree, maximal clique centrality, and stress. CRH and leptin (LEP) appeared in the top sets for betweenness, degree, maximal clique centrality (MCC), and stress, while cAMP response element-binding protein 1 (CREB1) and AKT serine/threonine kinase 1 (AKT1) ranked highly in selected shortest-path metrics. These findings identify proteins that are prominent within the constructed association network; they do not establish that these proteins are the only or dominant regulators in vivo.

**Figure 1:**
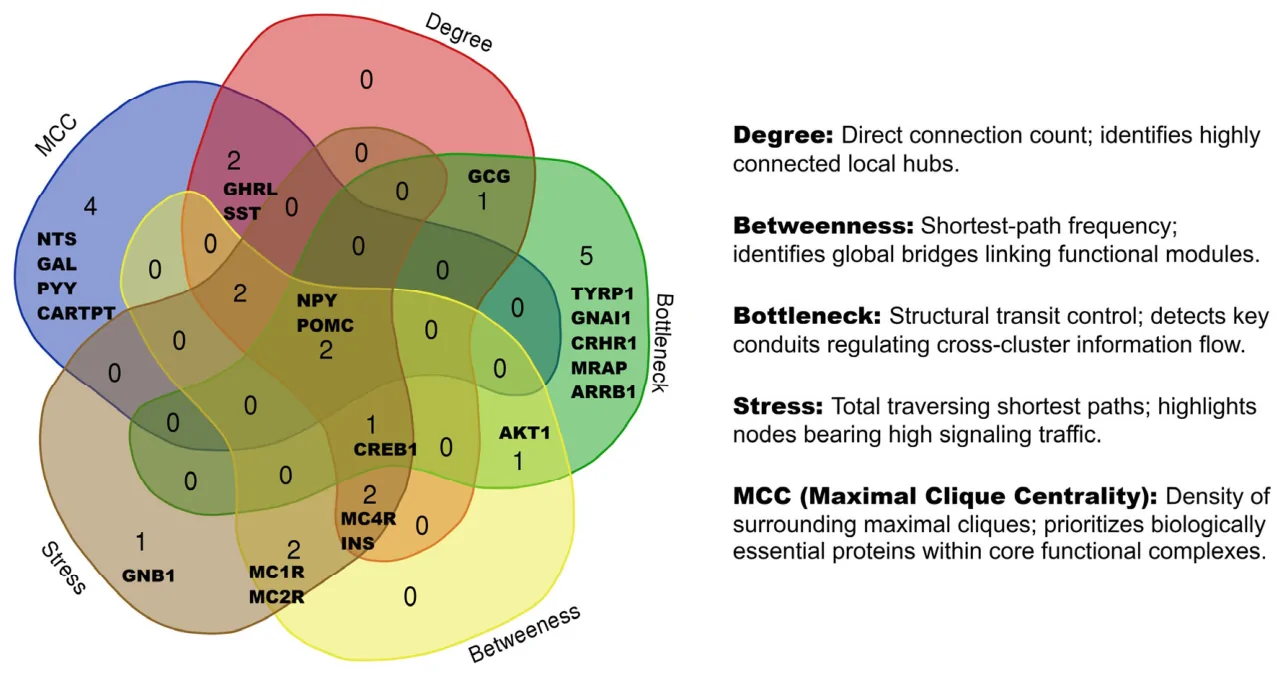
Identification of overlaping candidate genes evaluated by five complementary topological metrics implemented in the Cytoscape plugin cytoHubba that include Maximal Clique Centrality (MCC), Degree, Bottleneck, Betweenness, and Stress.

The pattern places appetite-regulating neuropeptides, HPA axis mediators, and intracellular signalling proteins within the same constructed network. This convergence informs the conceptual interpretation (Figure 1), but its biological importance requires testing in time-resolved experimental systems.

#### 2.2.3. Network Topology and Hub Identification

**Figure 2:**
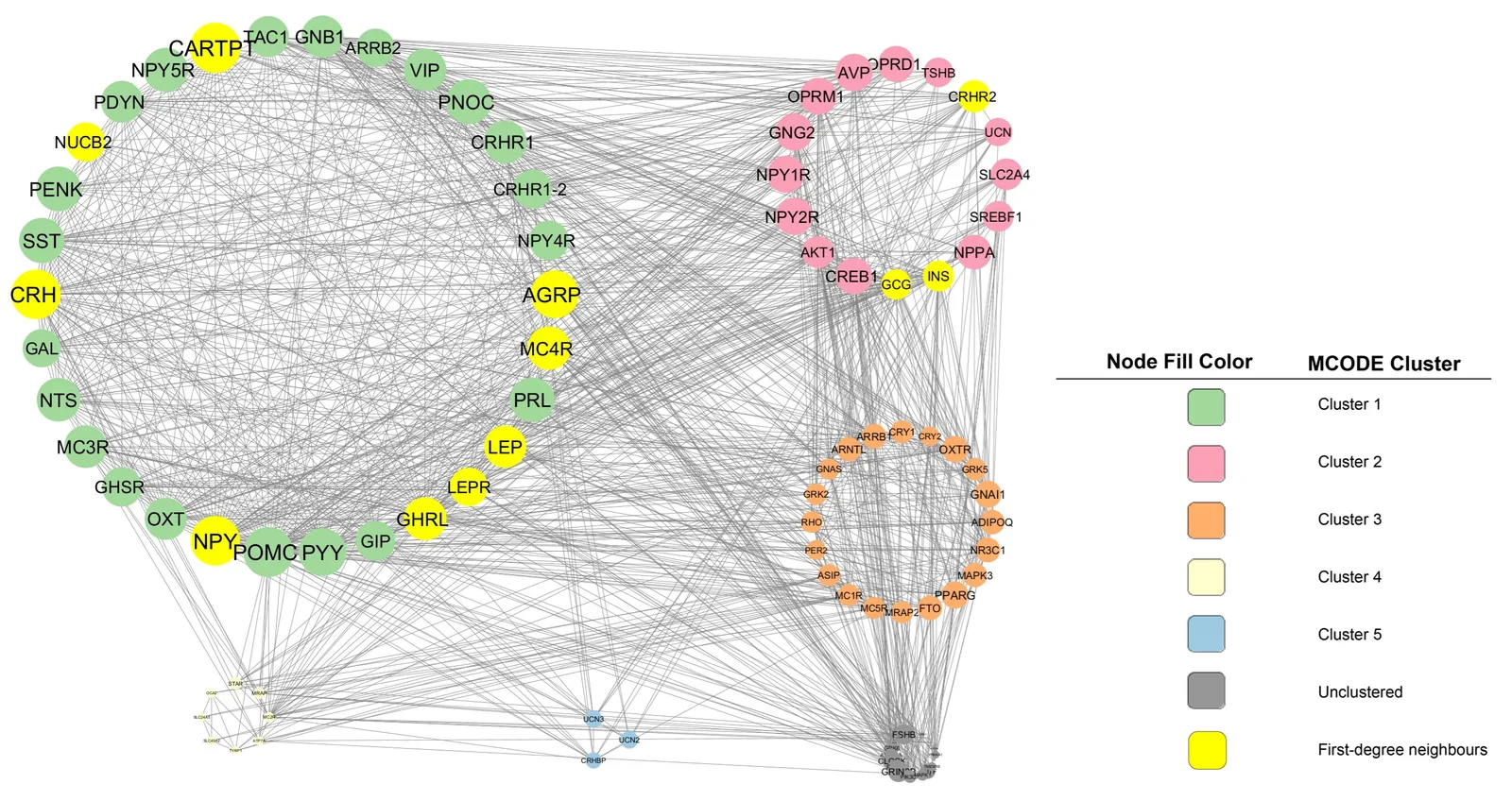
Functional protein-association network and modules derived from MCODE subnetwork analysis.

The Molecular Complex Detection (MCODE) plugin identified five densely connected modules. Cluster 1 had the highest score (20; 30 nodes and 290 edges), followed by Cluster 2 (score 6.667; 16 nodes and 50 edges), Cluster 3 (score 5.789; 20 nodes and 55 edges), Cluster 4 (score 3.143; 8 nodes and 11 edges), and Cluster 5 (score 3.000; 3 nodes and 3 edges). These clusters represent regions of high association density within the input network. Figure 2 shows the primary protein-association module (Cluster 1) derived from MCODE subnetwork analysis. The network highlights the primary functional cluster (score: 20.0; 30 nodes, 290 edges) extracted from the candidate dataset. Nodes highlighted in yellow denote the first-degree neighbours (direct incoming interactors/functional associations) of NUCB2 within the subnetwork, mapping its immediate topological sphere of interaction. Unhighlighted nodes represent closely connected second-degree partners involved in downstream neuropeptide signalling, energy homeostasis, and glucocorticoid regulation.

#### 2.2.4. Functional Enrichment Analysis

Gene Ontology (GO) and Kyoto Encyclopedia of Genes and Genomes (KEGG) enrichment of Cluster 1 showed strong representation of neuropeptide signalling and G-protein-coupled receptor signalling. This pattern is consistent with a network dominated by extracellular peptide communication and receptor-mediated signalling. It does not, however, distinguish direct physical binding from indirect functional association.

Cluster 1 was also enriched for adult feeding behaviour and positive regulation of cortisol secretion. The presence of ghrelin (GHRL), agouti-related neuropeptide (AGRP), NPY, POMC, and CRH within the same module supports functional overlap between appetite- and stress-related pathways in the assembled network. Because the candidate list was selected around these biological themes, the enrichment should be interpreted as convergence that prioritises hypotheses rather than as independent proof that the pathways form a single regulatory unit.

Enrichment for hormone activity and neuropeptide hormone activity further indicates that secreted signalling molecules are prominent in Cluster 1. The network alone cannot assign these associations to a specific anatomical nucleus or determine when they are active.

These database-derived associations were used as a complementary evidence-synthesis approach to contextualise molecular relationships identified from the published literature. The functional annotations of the proteins comprising Cluster 1 are summarised in Table 4.

## 3. Integrated Discussion and Translational Perspectives

Earlier reviews have focused mainly on the broad physiology of nesfatin-1, its metabolic actions, or its involvement in stress-related pathways [27,28,29]. The distinct contribution of the present review is a cross-dimensional synthesis that asks how reported effects vary across biological time, tissue level, and circadian alignment. Three findings define this comparison. First, PVN NUCB2/nesfatin-1 contributes to daily feeding patterning in nocturnal rodents. Second, central and pituitary studies place nesfatin-1 within stress-responsive pathways that can increase CRH, POMC, ACTH, and corticosterone. Third, a human adrenal cortical cell model shows a different local effect, with suppression of cortisol synthesis. The association network places NPY, POMC, CRH, LEP, GHRL, CREB1, and AKT1 within connected feeding- and stress-related pathways. Taken together, the evidence supports physiological overlap and tissue-dependent differences, but not a single uniform action of nesfatin-1 across the HPA axis.

Accordingly, this paper should be read as a gap-mapping review and a testable conceptual framework, not as evidence of a newly established regulatory mechanism. Its contribution is to organise daily feeding data, sleep findings, central HPA activation, adrenal inhibition, and network topology according to biological phase and site of action. This integration identifies three specific gaps: there is no established 24 h nesfatin-1 profile in humans, no direct characterisation of its phase relationship with cortisol, and no study showing how nesfatin-1 or its temporal relationship with cortisol behaves during shift work or other states of circadian misalignment.

### 3.1. Nesfatin-1 Within Daily Physiological Organisation

Collectively, the experimental findings suggest that hypothalamic NUCB2/nesfatin-1 may contribute to the daily organisation of feeding beyond its established anorexigenic effects. In particular, the phase-related changes in paraventricular NUCB2 expression and feeding responses following targeted manipulation suggest a potential role in maintaining feeding behaviour within the appropriate biological phase [9]. Nevertheless, these observations reflect regulation under conventional light–dark conditions and do not establish an endogenous circadian mechanism [5,9].

This interpretation requires two qualifications. A day-night difference is not by itself proof of an endogenous circadian rhythm, because light exposure, sleep, fasting duration, and recent feeding can all produce rhythmic changes. Demonstrating circadian control requires sampling under conditions that separate internal time from behaviour and environment. In addition, clock time cannot be translated directly from nocturnal rodents to humans. The relevant comparison is biological phase: the rodent light phase is usually the rest phase, whereas the human rest phase normally occurs at night. Current evidence therefore supports phase-related regulation in rodents, but it does not establish the timing or waveform of nesfatin-1 in people.

### 3.2. Feeding, Sleep, and the Biological Night

The original idea that nesfatin-1 might preserve nocturnal satiety without activating stress is attractive. Still, the available data do not yet support that conclusion. Central nesfatin-1 reliably suppresses food intake in rodents, including during the dark phase when they normally feed [15]. However, nesfatin-1 is also responsive to stress and can promote arousal-related effects. In the only direct sleep study identified, rapid-eye-movement (REM) sleep deprivation reduced nesfatin-1/NUCB2 expression. In contrast, central nesfatin-1 given at light onset reduced REM sleep, increased wakefulness, and increased sleep fragmentation [22]. Nesfatin-1 should therefore not be described as a sleep-consolidating signal on present evidence.

A more defensible interpretation is that nesfatin-1 lies at an interface where feeding state, vigilance, and stress are coordinated. The direction of its effect may change with brain region, dose, route, and timing. During ordinary feeding, PVN oxytocin and melanocortin pathways may mediate satiety. During acute stress, recruitment of CRH, noradrenergic, and serotonergic neurons may favour vigilance and HPA-axis activation [3]. This context-dependent view explains why reduced feeding does not necessarily indicate a calm or sleep-promoting state.

### 3.3. Tissue-Level Differences in Cortisol Regulation

The clearest tissue-specific contrast is the difference between central or pituitary effects and the direct adrenal-cell effect. In rats, intracerebroventricular nesfatin-1 activates CRH neurons and raises ACTH and corticosterone [3,8]. In murine corticotrophs, nesfatin-1 increases POMC abundance through adenylate cyclase, cAMP, protein kinase A (PKA), and CREB [18]. These findings support stimulation in the experimental hypothalamic and pituitary settings studied. By contrast, Nasri et al. (2024) found that nesfatin-1 reduced steroidogenic-enzyme expression and cortisol synthesis in human H295R adrenal cortical cells, also involving the cAMP/PKA/CREB pathway [10].

These results are not necessarily contradictory because the HPA axis is a multi-level system and the same peptide may have different actions in different cell types. One possible level-specific interpretation is that increased central or pituitary drive and local adrenal inhibition can coexist. However, the current studies were conducted in different species, tissues, and experimental conditions, so they cannot show that these effects operate together in vivo or form a physiological feedback mechanism. The adrenal evidence comes from one cell-line study, and no study has simultaneously measured nesfatin-1 activity at the relevant tissue levels, ACTH, and a full cortisol or corticosterone profile.

Theoretically, timing may contribute to the apparent divergence. Cortisol secretion reflects circadian drive, pulsatile ACTH release, recent awakening, stress exposure, and adrenal responsiveness. Experiments performed at one time point or after a pharmacological dose cannot determine whether nesfatin-1 changes the phase, amplitude, pulse pattern, or total output of the hormone. The unidentified receptor for nesfatin-1 adds further uncertainty because receptor abundance and downstream coupling may differ among hypothalamic neurons, corticotrophs, and adrenal cortical cells. Thus, the proposed central–peripheral distinction represents a conceptual interpretation of findings obtained from separate experimental systems, rather than evidence of an established physiological feedback mechanism.

Beyond its metabolic and neuroendocrine functions, NUCB2/nesfatin-1 has also been implicated in cancer biology. Experimental studies have reported that NUCB2/nesfatin-1 promotes cell proliferation, migration, and invasion in several tumour models, including gastric and endometrial carcinomas, while studies in breast and colorectal cancers have linked its activity to molecular pathways involved in tumour progression and metastasis [30,31,32]. However, these effects appear to be tissue- and context-dependent, with other studies reporting antiproliferative actions in certain cancer cell models [33]. These observations highlight the diverse biological functions of NUCB2/nesfatin-1 beyond feeding and neuroendocrine regulation. Nevertheless, the relevance of these cancer-associated mechanisms to circadian-feeding rhythms or HPA-axis regulation remains unclear and falls outside the primary scope of the present review.

### 3.4. The Network Analysis

The network analysis adds a hypothesis-prioritisation layer to the temporal and tissue-based synthesis. NPY and POMC were the most consistently high-ranking nodes, while CRH and LEP were also prominent. This pattern is compatible with experimental evidence that nesfatin-1 inhibits NPY neurons and recruits oxytocin-melanocortin signalling to reduce feeding [6,10]. The presence of CRH and GHRL within the enriched module identifies candidates through which stress and hunger signals might converge. Still, it does not demonstrate that convergence occurs in a specific tissue or at a specific biological time.

Within Cluster 1, the co-occurrence of NUCB2 with feeding-related neuropeptides and HPA-axis-associated signalling molecules suggests a potential molecular interface between metabolic and stress-related pathways. This network organisation provides a basis for prioritising candidate molecules for further experimental investigation.

Nevertheless, this network architecture must be interpreted with appropriate scientific restraint. Because STRING incorporates both direct physical interactions and predicted functional associations, an edge between NUCB2 and its yellow-highlighted first-degree neighbours denotes biological association rather than proven physical complex formation, direct enzymatic action, or directional causality in vivo. Furthermore, a dense subnetwork cannot pinpoint specific anatomical loci (e.g., PVN vs. arcuate nucleus vs. adrenal cortex) nor confirm whether these associations are active simultaneously or in a phase-dependent manner across the 24 h cycle. In addition, because the initial seed list was purposely enriched with feeding, circadian, and HPA-axis targets, pathway enrichment in these domains is partially expected.

The primary value of Cluster 1 and its direct NUCB2 neighbourhood is therefore to supply a prioritised candidate map, specifically highlighting NPY, POMC, CRH, GHRL, CREB1, and AKT1 for future time-resolved functional validation. The network identifies potential nodes of convergence, but only empirical chronophysiological studies incorporating time-series sampling can determine their temporal dynamics and physiological directionality.

### 3.5. Circadian Misalignment as an Unresolved Domain

Cortisol disruption in shift work is well documented, but its form is not uniform. Studies differ in shift schedule, number of consecutive nights, sleep timing, light exposure, and cortisol sampling. A scoping review concluded that night and irregular shift work can alter cortisol levels, rhythm, and the cortisol awakening response, while also emphasising major methodological heterogeneity [6]. This evidence establishes disruption of the stress axis but does not show that nesfatin-1 is involved.

Against this background, the potential behaviour of nesfatin-1 during circadian misalignment warrants consideration. If nesfatin-1 normally helps restrict feeding during the rest phase, a shift in its timing or a reduction in its amplitude could contribute to eating during the biological night. Alternatively, if stress-responsive nesfatin-1 remains aligned with internal circadian time while behaviour shifts rapidly, its relationship with cortisol and meals could become mistimed. These are competing hypotheses. Neither should be presented as an established mechanism until nesfatin-1, cortisol, sleep, light exposure, and meal timing are measured together.

### 3.6. Strengths and Limitations of the Review

The principal strength of this review lies in its integrated critical evaluation of biological timing, experimental site of action, and measurement type, rather than a descriptive summary of existing findings. By comparing evidence across different experimental models, the review highlights tissue-specific responses, apparent contradictions between central and peripheral actions of nesfatin-1, and limitations in interpreting daily variation as evidence of endogenous circadian rhythmicity. The narrative synthesis and protein-association analysis are kept conceptually distinct: the former critically evaluates reported observations, whereas the latter prioritises candidate molecular associations for future experimental investigation.

The evidence was heterogeneous in biological model, intervention, and outcome. Circulating peptide concentrations, hypothalamic transcript or protein measurements, neuronal activation, meal microstructure, plasma glucocorticoids, and adrenal-cell cortisol output address related but non-equivalent processes. In particular, precursor expression cannot be equated with mature peptide secretion, and circulating concentrations do not directly quantify secretion rate. Central administration of full-length nesfatin-1, administration of its active fragment, and exposure to NUCB1-derived nesfatin-1-like peptide also represent different experimental contrasts. These differences limit direct comparison and preclude a single integrated effect estimate from the present extraction [3,10,11,14,16,22].

Evidence for endogenous circadian regulation was particularly limited. Sparse sampling, comparisons performed during exercise or dietary manipulation, and sleep-deprivation protocols did not separate endogenous timing from behavioural and environmental influences. Differences in feeding state further complicated interpretation of the morning–night exercise comparison. The human exercise evidence was restricted to young men, while the mechanistic feeding and HPA findings relied on animal experiments or a transformed adrenal cell line. Accordingly, transferability to women, older adults, clinical populations, and occupational shift workers remains uncertain [10,11,12,13,16,22].

As a narrative review incorporating an exploratory protein-association analysis, the present work does not provide original experimental findings or establish a causal or endogenous circadian relationship between nesfatin-1 and cortisol. Narrative synthesis was employed because the biological models, interventions, and outcomes were too heterogeneous for meaningful quantitative pooling. Although this approach can identify patterns, contradictions, and knowledge gaps, it cannot estimate an overall effect size. Furthermore, the synthesis was limited to the selected English-language original articles included in the extraction table. The complementary protein-association analysis is exploratory, and its findings are sensitive to the selected input genes, available database annotations, confidence threshold, and topology metrics. These associations do not constitute independent mechanistic validation and require validation through expression, perturbation, and time-series experiments. Accordingly, the proposed conceptual framework should be interpreted as a basis for future hypothesis testing rather than evidence of an established physiological interaction.

### 3.7. Future Research Priorities

The priority is a controlled 24 h human study with repeated nesfatin-1 and cortisol sampling, standardised meals, documented posture and activity, and objective sleep and light measurements. Melatonin onset should be included as a marker of internal circadian phase. Analysis should estimate each participant’s timing of peak, amplitude, and the time interval between nesfatin-1 and cortisol rhythms. Assays should clearly state whether they detect intact NUCB2, nesfatin-1, or related immunoreactivity.

A second priority is a within-person shift-work design comparing day work, night work, and recovery days. Sampling by both clock time and biological time would distinguish a true phase shift from changes caused by sleep, meals, or work stress. Sex, menstrual status, chronotype, adiposity, sleep debt, and recent food intake should be treated as planned covariates rather than post hoc explanations.

Mechanistic studies should then test whether the level-specific pattern proposed by this review is reproducible and causal. Timed experiments should measure CRH, ACTH, and adrenal steroid output within the same protocol after nesfatin-1 manipulation, with sampling anchored to biological phase. Cell-specific knockdown and receptor-identification studies could then determine whether differences among hypothalamic, pituitary, and adrenal responses arise from distinct cellular signalling. Finally, time-resolved perturbation of the network-prioritised candidates should test whether NPY, POMC, CRH, GHRL, CREB1, or AKT1 mediates any observed relationship among nesfatin-1, feeding, and glucocorticoid output. Until such experiments are performed, these proteins should be regarded as candidates rather than established temporal mediators.

**Figure 3:**
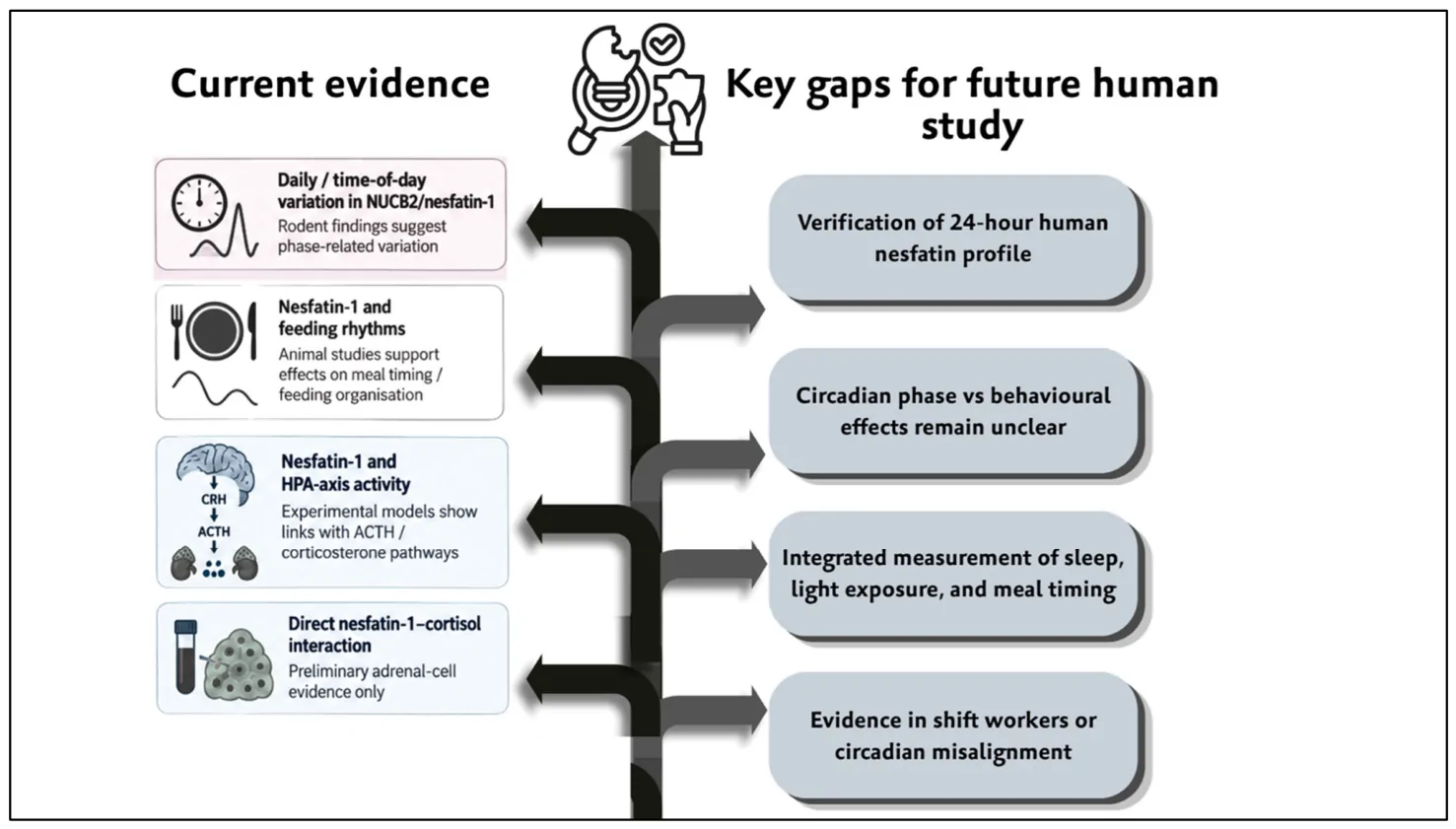
Conceptual framework of potential human studies on NUCB2/nesfatin-1.

Figure 3 illustrates the conceptual framework linking current experimental evidence on NUCB2/nesfatin-1 with unresolved chronophysiological gaps. Despite evidence of its involvement in feeding regulation and HPA-axis activity, the 24 h profile of nesfatin-1, its temporal relationship with cortisol, and its response to circadian misalignment remain poorly characterised in humans. These gaps highlight the need for time-resolved human studies integrating hormonal, circadian, and behavioural measurements, particularly among shift workers.

## 4. Review and Analytical Approach

### 4.1. Literature Search Strategy

Literature was identified through exploratory, topic-focused searches of PubMed/MEDLINE, Scopus, and Web of Science, covering publications relevant to NUCB2/nesfatin-1, biological timing, feeding regulation, HPA-axis activity, and cortisol synthesis. Search terms included combinations of “nesfatin-1”, “NUCB2”, “circadian”, “diurnal”, “feeding”, “cortisol”, “corticosterone”, “HPA axis”, “sleep”, and “shift work”. Relevant original research articles were selected purposively according to their contribution to the predefined physiological domains of this narrative review. Reference lists of relevant publications were also examined to identify additional studies. Targeted literature searches undertaken during manuscript revision further informed the evidence synthesis.

The literature-identification process was intended to support a critical narrative review. Consequently, formal systematic screening procedures and comprehensive study-selection counts were not maintained. The findings and identified evidence gaps are therefore interpreted within the scope of the literature examined.

### 4.2. Eligibility Criteria

Studies were eligible for inclusion in the primary evidence synthesis if they were original research articles involving human participants, animal models, or in vitro experimental systems. Studies were considered relevant if they investigated NUCB2/nesfatin-1 in relation to feeding regulation, biological timing, sleep, HPA-axis activity, or glucocorticoid synthesis. All review-type publications, including narrative reviews, systematic reviews, scoping reviews, and meta-analyses, were excluded from the primary evidence synthesis. Editorials, commentaries, case reports, and non-peer-reviewed publications were also excluded. Review publications could be consulted for background information and reference identification but were not considered primary evidence.

### 4.3. Evidence Synthesis

Eligible evidence was organised into five thematic domains:

1. Daily and state-dependent variation in NUCB2/nesfatin-1
2. Nesfatin-1 and feeding rhythms
3. Nesfatin-1 and HPA-axis activity
4. Direct nesfatin-1–cortisol interaction
5. Nesfatin-1 in shift workers

Because the eligible studies differed substantially in species, experimental model, exposure, and outcome, findings were synthesised narratively. The purpose was to map converging and conflicting evidence and to identify questions that require direct temporal testing; no pooled effect estimate was calculated.

### 4.4. Protein-Association Analysis

To explore functional associations relevant to nesfatin-1, feeding, and HPA axis regulation, an in silico protein-association analysis was performed alongside the narrative synthesis. A literature-derived seed list included NUCB2 and proteins linked to circadian regulation, satiety signalling, and cortisol synthesis. This list was expanded using first- and second-shell associations returned by STRING version 12.0, after which duplicate entries were removed. Because STRING includes both direct physical interactions and indirect functional associations, network edges were interpreted as hypothesis-generating evidence of biological association, rather than proof of molecular binding, causal mediation, timing, or anatomical co-localisation [4].

### 4.5. Functional Protein-Association Network Construction

The identified targets were queried in STRING version 12.0 with the organism restricted to *Homo sapiens*. A minimum combined interaction score of 0.700 was applied to retain high-confidence associations.

### 4.6. Network Visualisation and Hub Node Identification

The resulting network was imported into Cytoscape version 3.10.3. Degree, betweenness, stress, bottleneck, and maximal clique centrality were calculated with cytoHubba to rank highly connected or strategically positioned nodes. These rankings were used to prioritise candidates for biological interpretation and experimental validation, not to infer causal importance [2].

### 4.7. Module Analysis via MCODE

Densely connected subnetworks were identified with the MCODE plugin in Cytoscape [1]. Standard parameters were applied: degree cutoff = 2, node score cutoff = 0.2, K-core = 2, and maximum depth = 100.

### 4.8. Functional and Pathway Enrichment Analysis

To determine the biological significance of the specific MCODE-derived modules, the gene lists were submitted to the DAVID database. Enrichment analysis was performed using the KEGG pathway database, focusing on biological functions related to neuropeptide signalling, feeding behaviour, and HPA-axis regulation. Pathways with a False Discovery Rate (FDR) < 0.05 were considered statistically significant.

## 5. Conclusions

**Figure 4:**
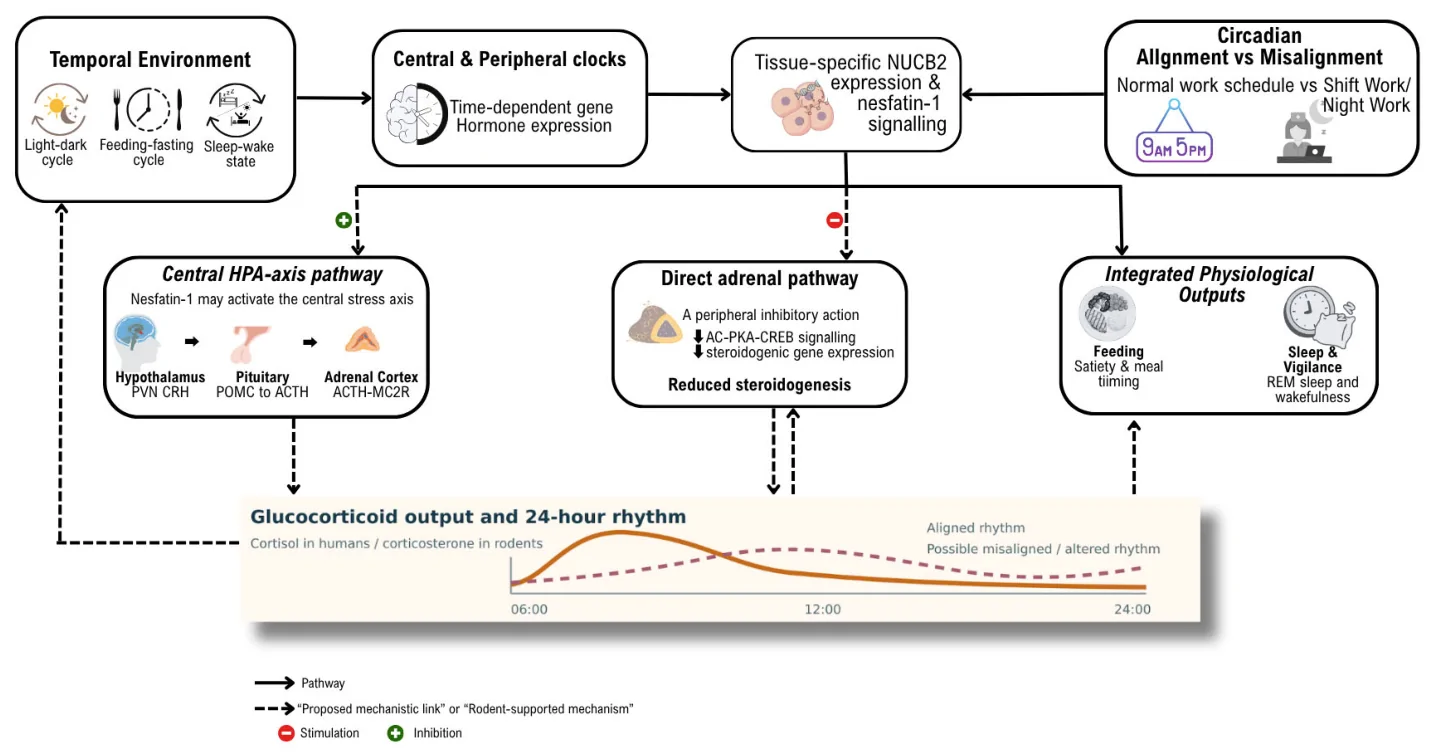
Conceptual framework of chronophysiological model linking NUCB2/nesfatin-1, the feeding regulation, sleep–wake organisation, and HPA-axis activity.

The reviewed experiments identify context-dependent effects of NUCB2/nesfatin-1 on feeding and HPA-related outcomes, but do not establish a coordinated human nesfatin-1–cortisol rhythm. Central rodent experiments and adrenal-cell experiments test different biological contrasts and cannot demonstrate an integrated feedback circuit (Figure 4). The constructed association network supplies candidates for future tests, not independent validation of the framework. As the network was constructed from biologically selected candidates, its pathway enrichment findings should be interpreted as hypothesis-generating rather than independent evidence of nesfatin-1-mediated coordination between feeding and cortisol regulation.

The conceptual model in Figure 3 suggests that circadian alignment, feeding, and sleep-wake timing may influence tissue-specific nesfatin-1 signalling and its interaction with the HPA axis. Nesfatin-1 could affect glucocorticoid production through opposing central stimulatory and peripheral inhibitory pathways, with potential consequences for feeding, sleep, and vigilance. However, although cortisol disruption during circadian misalignment is well established, the 24 h rhythm of nesfatin-1 and its temporal relationship with cortisol remain unclear. Therefore, these pathways should be considered proposed mechanisms that require validation through controlled 24 h human studies under aligned and misaligned conditions.

## Figures and Tables

**Table 1:** The representative evidence and the main strength and limitation within each domain of interest.

Domain	Representative Evidence	Strength and Limitation
Daily and state-dependent variation in NUCB2/nesfatin-1	Rodent PVN expression and circulating measurements, supported by sleep-related rodent and time-of-day human exercise studies [11,12,13].	Limitation: direct PVN daily variation demonstrated in rats, but other evidence is context-dependent; no complete human 24 h basal profile or demonstrated endogenous circadian rhythm in these studies.
Nesfatin-1 and feeding rhythms	Rodent PVN manipulations support light-phase feeding suppression [4,5], while central nesfatin-1 or its 30–59 fragment alters meal size and/or spacing [14,15,16]. Other included experiments primarily demonstrate altered intake quantity rather than feeding timing.	Strength: complementary endogenous PVN manipulations and meal-pattern measurements support phase-dependent feeding suppression and altered meal spacing. Limitation: meal-size changes concern quantity rather than timing; fragment effects depend on metabolic condition, and first-meal latency was unchanged in the full-length mouse experiment. Light–dark intake differences and acute post-injection meal-spacing effects do not establish endogenous circadian control. One particular study in the existing evidence table was verifiable only at the original-abstract level and not treated as confirmed evidence [17].
Nesfatin-1 and HPA axis activity	Central nesfatin-1 administration increased plasma ACTH and corticosterone in rats [3,8], with PVN CRH-neuron activation demonstrated by Yoshida et al. [3]. In murine AtT-20 corticotrophs, nesfatin-1 stimulated POMC synthesis with pharmacological evidence implicating adenylate cyclase/PKA/CREB signalling [18].	Strength: direct endocrine responses in vivo, supported by CRH-neuron activation and inhibitor-sensitive pituitary POMC synthesis. Limitation: exogenous-peptide responses do not establish endogenous nesfatin-1 necessity; CRH-neuron activation does not itself establish CRH mediation of the hormonal response. POMC induction must not be equated with demonstrated ACTH secretion.
Direct nesfatin-1–cortisol interaction	Human H295R experiments support direct cortisol suppression by nesfatin-1/NLP, whereas rodent, goldfish, and pituitary-cell studies indicate upstream HPA/HPI-axis stimulation; earlier adrenal-cell work addressed proliferation/apoptosis rather than cortisol regulation [3,8,10,18,19,20].	Strength: direct measurement of cortisol in an isolated human adrenal-derived cell model distinguishes the adrenal effect from central/pituitary regulation. Limitations: only one direct adrenal cortisol study was identified within the specified publication window; functional evidence is restricted to a transformed H295R line, without identified primary human adrenal replication. Cortisol was tested at one peptide concentration with a limited time course; steroidogenic mRNA changes do not demonstrate enzyme activity, and transcript exposure times differ between Results and Methods. Central, pituitary, and whole-animal findings do not establish direct adrenal inhibition or reduced circulating glucocorticoids in humans.
Nesfatin-1 in shift workers	A healthcare-worker thesis reported no shift–day difference in serum nesfatin-1, but a positive stress–nesfatin-1 association in male shift workers [21].	Strength: direct occupational sampling with daytime controls. Limitations: cross-sectional, abstract-level grey literature; assay and circadian-phase details unverified, with no serial hormone profile described.

**Table 2:** Characteristics of studies examining nesfatin-1 in circadian and HPA contexts.

Reference	Model or Sample	Main Findings	Domain	Gap
Algul et al. (2017) [12]	Young trained and untrained men; morning and night aerobic-exercise sessions.	Serum nesfatin-1 increased after night-time but not morning exercise. Fasting/fed differences confound the time-of-day comparison; this is not a basal daily secretion profile.	1. Daily regulation of nesfatin-1—adjacent exercise/time-of-day evidence	Basal daily regulation remains unestablished: exercise responses cannot separate clock-time effects from fasting/fed conditions.
Mohan et al. (2014) [11]	In vitro: mouse stomach ghrelinoma MGN3-1 cells treated with glucose, fatty acids, or amino acids. In vivo: male C57BL/6 mice fed high-fat, high-carbohydrate, or high-protein diets chronically; a separate high-fat bolus-gavage comparison is also described.	In MGN3-1 cells, glucose and L-tryptophan increased NUCB2 expression; L-tryptophan increased nesfatin-1 secretion, while oleic acid reduced NUCB2 expression. In mice, chronic diets and high-fat gavage altered tissue NUCB2 and serum nesfatin-1 at sampled times; the comparisons did not establish a within-group daily rhythm.	1. Daily regulation of nesfatin-1—time-specific dietary comparisons, not a demonstrated rhythm	A within-group daily waveform and endogenous circadian regulation remain unestablished; time-specific dietary comparisons do not establish rhythmic secretion.
Ozcelik et al. (2018) [13]	Young trained men; morning, afternoon, and night soccer matches.	In 20 trained young men, serum nesfatin-1 increased significantly after morning and night soccer matches, but not after afternoon matches. Baseline levels did not differ significantly across sessions. Because sampling followed exercise, the findings do not establish an endogenous daily nesfatin-1 rhythm.	1. Daily regulation of nesfatin-1—adjacent exercise/time-of-day evidence	Endogenous daily regulation remains unestablished: pre/post-exercise comparisons do not characterize a spontaneous daily nesfatin-1 profile.
Vas et al. (2013) [22]	Rats: 72 h REM-sleep deprivation followed by rebound sleep, with time-matched controls; separate intracerebroventricular nesfatin-1 or saline administration with EEG analysis.	In rats, REM-sleep deprivation reduced hypothalamic nesfatin-1/NUCB2 mRNA and protein, while rebound sleep restored levels; intracerebroventricular nesfatin-1 altered sleep and wakefulness. The limited daytime controls did not establish a spontaneous daily rhythm.	1. Daily regulation of nesfatin-1—adjacent sleep-state evidence	A full-day expression profile and endogenous rhythmicity remain unestablished; sleep-deprivation and rebound responses do not establish circadian regulation.
Dong et al. (2014) [24]	Rats: in vivo DVC nesfatin-1 administration and food-intake/body-weight measures; DVC glucosensing neurons assessed in slice electrophysiology.	In rats, DVC nesfatin-1 reduced food intake over 5 h and body-weight gain with chronic administration; it also altered DVC glucosensing-neuron activity. Food intake was examined in the dark phase, but feeding rhythms were not tested.	2. Nesfatin-1 and feeding rhythms—dark-phase intake, not rhythm analysis	Redistribution of feeding across the day remains unestablished: reduced dark-phase cumulative intake is not evidence of altered feeding rhythmicity.
Goebel et al. (2011; online 2010) [14]	Adult male C57BL/6 mice; ad libitum-fed. Food intake and meal patterns were monitored after central or peripheral nesfatin-1 administration; brain immunohistochemistry was also performed (*n* = 3).	In adult male C57BL/6 mice, intracerebroventricular nesfatin-1 reduced dark-phase food intake, with delayed onset, smaller meals, and longer inter-meal intervals; peripheral administration did not alter feeding. This supports a central anorexigenic effect during nocturnal feeding.	2. Nesfatin-1 and feeding rhythms—meal microstructure	Whether endogenous nesfatin-1 controls daily feeding phase remains unestablished; acute central administration during nocturnal feeding demonstrates meal-pattern effects.
Prinz et al. (2015) [16]	Male Sprague–Dawley rats on standard diet and rats with diet-induced obesity after high-fat feeding; central (intracerebroventricular) and peripheral (intraperitoneal) nesfatin-1(30–59) interventions.	Central nesfatin-1(30–59) reduced intake through smaller meals in normal-weight rats and reduced meal frequency with longer inter-meal intervals in diet-induced-obese rats. Peripheral injection had no feeding effect.	2. Nesfatin-1 and feeding rhythms—meal microstructure	Whether endogenous full-length nesfatin-1 regulates daily feeding rhythms remains unestablished by pharmacological fragment administration and meal-microstructure responses.
Toru et al. (2015) [25]	28 two-month-old male Wistar Albino rats (200–250 g), assigned to laparotomy, ischemia/reperfusion, nesfatin-1 plus laparotomy, and nesfatin-1 plus ischemia/reperfusion groups (*n* = 7 each).	Chronic peripheral nesfatin-1 lowered plasma cortisol during intestinal ischemia/reperfusion versus injury alone, but increased cortisol after laparotomy versus laparotomy alone. Systemic responses do not isolate adrenal action; the authors identify measuring cortisol rather than corticosterone in rats as a limitation.	3. Nesfatin-1 and HPA axis activity—systemic cortisol response, not isolated adrenal action	The site and mechanism of the systemic cortisol response remain unresolved; direct adrenal action is not isolated, and cortisol rather than corticosterone was measured in rats.
Yoshida et al. (2010) [3]	Male Sprague-Dawley rats (7–8 weeks old); isolated hypothalamic PVN neurons were also used for calcium-response experiments.	In male rats, central nesfatin-1 activated PVN CRH and other stress-related neurons and increased plasma ACTH and corticosterone, supporting central HPA-axis activation. Corticosterone—not cortisol—was measured; no direct adrenal nesfatin-1 effect was tested.	3. Nesfatin-1 and HPA axis activity	Direct adrenal effects on cortisol remain untested; central CRH-neuron activation and ACTH/corticosterone responses do not establish a direct nesfatin-1–cortisol mechanism.
Nasri et al. (2024) [10]	Human H295R adrenocortical carcinoma cells; accompanying wild-type and Nucb1-knockout mouse experiments.	Nesfatin-1 and NLP reduced cellular cortisol content and forskolin-stimulated cortisol release, alongside reduced steroidogenic transcripts and CREB phosphorylation. Direct action is supported in isolated cells; the mouse arms did not establish direct in vivo adrenal inhibition.	4. Direct nesfatin-1–cortisol interaction; 3. Nesfatin-1 and HPA axis activity—accompanying gene-expression evidence	Physiological relevance in primary human adrenal cells and direct adrenal inhibition in vivo remain unestablished by the transformed-cell and accompanying mouse experiments.

**Table 3:** Genes listed for the in silico analysis.

*NPY*	*NUCB2*	*OXT*	*POMC*	*CRH*	*LEP*	*MC1R*	*MC2R*	*MC3R*	*MC4R*
*MC5R*	*MRAP*	*OPRM1*	*PDYN*	*CRHBP*	*CRHR1*	*CRHR1-2*	*CRHR2*	*GNAS*	*UCN*
*UCN2*	*UCN3*	*GAL*	*NPY1R*	*NPY5R*	*GHRL*	*SST*	*NPY2R*	*PYY*	*NPY4R*
*GCG*	*PRL*	*ADIPOQ*	*AGRP*	*LEPR*	*INS*	*CNTF*	*NTS*	*TAC1*	*AVP*
*AVPR1A*	*OXTR*	*PNOC*	*OPRD1*	*PENK*	*GRIN2B*	*GRIN2A*	*GRIN2D*	*GRK5*	*GRK6*
*GRK2*	*ARRB1*	*ARRB2*	*GNAI1*	*MGRN1*	*SLC24A5*	*SLC45A2*	*ASIP*	*OCA2*	*TYRP1*
*GNB1*	*MRAP2*	*NPPA*	*FSHB*	*GIP*	*VIP*	*TSHB*	*ATP7A*	*GHSR*	*CTSZ*
*KCTD15*	*FTO*	*TMEM18*	*RHO*	*MT2A*	*GNG2*	*FGD1*	*FGD3*	*GPR12*	*CARTPT*
*STAR*	*CLOCK*	*ARNTL*	*PER1*	*CRY1*	*PRKAA*	*AKT1*	*MAPK1*	*CREB1*	*PPARG*
*SREBF1*	*PIK3CA*	*SLC2A4*	*NR3C1*	*CACNA1C*	*KCNA1*	*PER2*	*CRY2*	*PRKAA2*	*MAPK3*

**Table 4:** Database-derived functional annotations and enrichment terms for proteins in Cluster 1.

Term	Description	Proteins	*p*-Value	FDR
CC_GO:0005576	Extracellular region	OXT, CARTPT, PRL, PDYN, NTS, GIP, POMC, PYY, GAL, SST, LEP, NPY, PNOC, PENK, CRH, LEPR, AGRP, GHRL, VIP, TAC1, NUCB2	1.8 × 10^−12^	9.6 × 10^−11^
CC_GO:0043025	Neuronal cell body	GAL, SST, PNOC, PENK, CRH, AGRP, PDYN, TAC1	5.3 × 10^−7^	7.1 × 10^−6^
CC_GO:0098992	Neuronal dense core vesicle	SST, NPY, OXT, PDYN, TAC1	3.3 × 10^−8^	5.8 × 10^−7^
CC_GO:0030141	Secretory granule	POMC, GAL, OXT, CARTPT	4.1 × 10^−4^	0.003
BP_GO:0007218	Neuropeptide signalling pathway	NPY5R, NPY4R, CARTPT, PDYN, NTS, POMC, PYY, GAL, NPY, PNOC, PENK, AGRP, TAC1	3.0 × 10^−20^	1.2 × 10^−17^
BP_GO:0007186	G-protein-coupled receptor signalling pathway	MC4R, GHSR, MC3R, NPY5R, NPY4R, SST, GNB1, GHRL, CARTPT, VIP, CRHR1	9.7 × 10^−7^	4.2 × 10^−5^
BP_GO:0008343	Adult feeding behaviour	GHSR, LEP, NPY, AGRP, GHRL, CARTPT	1.0 × 10^−13^	2.0 × 10^−10^
BP_GO:0051464	Positive regulation of cortisol secretion	GAL, CRH, GHRL	1.2 × 10^−5^	3.1 × 10^−4^
MF_GO:0005179	Hormone activity	POMC, PYY, GAL, SST, LEP, NPY, CRH, GHRL, OXT, PRL, VIP, GIP	1.3 × 10^−17^	4.8 × 10^−16^
MF_GO:0005184	Neuropeptide hormone activity	PYY, GAL, NPY, PNOC, PENK, CRH, AGRP, OXT, CARTPT, VIP, NTS	2.9 × 10^−22^	2.2 × 10^−20^
hsa04024	cAMP signalling pathway	POMC, GHSR, SST, NPY, CRH, GHRL, OXT, VIP, CRHR1, GIP	4.6 × 10^−9^	7.3 × 10^−8^

Note: Protein–protein interaction networks and preliminary interactor mappings were retrieved from the STRING database (version 12.0) [26]. Downstream Gene Ontology (GO) terms and Kyoto Encyclopedia of Genes and Genomes (KEGG) pathway enrichment analyses for MCODE-derived modules (including Cluster 1) were executed using the Database for Annotation, Visualization and Integrated Discovery (DAVID, v[e.g., 2021/2023]). Statistical significance is reported as raw *p*-values (Fisher’s exact test) and adjusted *p*-values corrected for multiple hypothesis testing using the Benjamini–Hochberg False Discovery Rate (FDR).

## Data Availability

No new data were created or analyzed in this study. Data sharing is not applicable to this article.

## Abbreviations

The following abbreviations are used in this manuscript:

ACTH	adrenocorticotropic hormone
AGRP	agouti-related neuropeptide
AKT1	AKT serine/threonine kinase 1
cAMP	cyclic adenosine monophosphate
CREB	cAMP response element-binding protein
CRH	corticotropin-releasing hormone
FDR	false discovery rate
GHRL	ghrelin
HPA	hypothalamic–pituitary–adrenal
LEP	leptin
MCC	maximal clique centrality
NPY	neuropeptide Y
NUCB2	nucleobindin-2
PKA	protein kinase A
POMC	proopiomelanocortin
PVN	paraventricular nucleus
REM	rapid eye movement
SON	supraoptic nucleus

## References

[1] Bader G.D., Hogue C.W.V. (2003). An automated method for finding molecular complexes in large protein interaction networks. BMC Bioinform..

[2] Chin C.-H., Chen S.-H., Wu H.-H., Ho C.-W., Ko M.-T., Lin C.-Y. (2014). cytoHubba: Identifying hub objects and sub-networks from complex interactome. BMC Syst. Biol..

[3] Yoshida N., Maejima Y., Sedbazar U., Ando A., Kurita H., Damdindorj B., Takano E., Gantulga D., Iwasaki Y., Kurashina T. (2010). Stressor-responsive central nesfatin-1 activates corticotropin-releasing hormone, noradrenaline and serotonin neurons and evokes hypothalamic-pituitary-adrenal axis. Aging.

[4] Sedbazar U., Maejima Y., Nakata M., Mori M., Yada T. (2013). Paraventricular NUCB2/nesfatin-1 rises in synchrony with feeding suppression during early light phase in rats. Biochem. Biophys. Res. Commun..

[5] Nakata M., Gantulga D., Santoso P., Zhang B., Masuda C., Mori M., Okada T., Yada T. (2016). Paraventricular NUCB2/Nesfatin-1 Supports Oxytocin and Vasopressin Neurons to Control Feeding Behavior and Fluid Balance in Male Mice. Endocrinology.

[6] Grosser L., Knayfati S., Yates C., Dorrian J., Banks S. (2022). Cortisol and shiftwork: A scoping review. Sleep Med. Rev..

[7] Kohno D., Nakata M., Maejima Y., Shimizu H., Sedbazar U., Yoshida N., Dezaki K., Onaka T., Mori M., Yada T. (2008). Nesfatin-1 Neurons in Paraventricular and Supraoptic Nuclei of the Rat Hypothalamus Coexpress Oxytocin and Vasopressin and Are Activated by Refeeding. Endocrinology.

[8] Könczöl K., Bodnár I., Zelena D., Pintér O., Papp R.S., Palkovits M., Nagy G.M., Tóth Z.E. (2010). Nesfatin-1/NUCB2 may participate in the activation of the hypothalamic–pituitary–adrenal axis in rats. Neurochem. Int..

[9] Maejima Y., Sedbazar U., Suyama S., Kohno D., Onaka T., Takano E., Yoshida N., Koike M., Uchiyama Y., Fujiwara K. (2009). Nesfatin-1-Regulated Oxytocinergic Signaling in the Paraventricular Nucleus Causes Anorexia through a Leptin-Independent Melanocortin Pathway. Cell Metab..

[10] Nasri A., Sands J., Unniappan S. (2024). Suppressive action of nesfatin-1 and nesfatin-1-like peptide on cortisol synthesis in human adrenal cortex cells. Sci. Rep..

[11] Mohan H., Ramesh N., Mortazavi S., Le A., Iwakura H., Unniappan S. (2014). Nutrients differentially regulate nucleobindin-2/nesfatin-1 in vitro in cultured stomach ghrelinoma (MGN3-1) cells and in vivo in male mice. PLoS ONE.

[12] Algul S., Ozdenk C., Ozcelik O. (2017). Variations in leptin, nesfatin-1 and irisin levels induced by aerobic exercise in young trained and untrained male subjects. Biol. Sport.

[13] Ozcelik O., Algul S., Yılmaz B. (2018). Nesfatin-1 and irisin levels in response to the soccer matches performed in morning, afternoon and at night in young trained male subjects. Cell. Mol. Biol..

[14] Goebel M., Stengel A., Wang L., Taché Y. (2011). Central nesfatin-1 reduces the nocturnal food intake in mice by reducing meal size and increasing inter-meal intervals. Peptides.

[15] Stengel A., Goebel M., Wang L., Rivier J., Kobelt P., Mönnikes H., Lambrecht N.W.G., Taché Y. (2009). Central Nesfatin-1 Reduces Dark-Phase Food Intake and Gastric Emptying in Rats: Differential Role of Corticotropin-Releasing Factor2 Receptor. Endocrinology.

[16] Prinz P., Teuffel P., Lembke V., Kobelt P., Goebel-Stengel M., Hofmann T., Rose M., Klapp B.F., Stengel A. (2015). Nesfatin-1_30_–_59_ injected intracerebroventricularly differentially affects food intake microstructure in rats under normal weight and diet-induced obese conditions. Front. Neurosci..

[17] Xue R.M., Wang M., Leng H., Sun X.R., Xu L. (2018). Effects of Nesfatin-1 on the Metabolism of Glycolipids in the Lateral Ventricle of Rats and its Relationship with the Expression of AMPK in Hypothalamus. Int. J. Sci..

[18] Nasri A., Unniappan S. (2021). Nucleobindin-derived nesfatin-1 and nesfatin-1-like peptide stimulate pro-opiomelanocortin synthesis in murine AtT-20 corticotrophs through the cAMP/PKA/CREB signaling pathway. Mol. Cell. Endocrinol..

[19] Pham V., Pemberton J.G., Chang J.P., Blanco A.M., Nasri A., Unniappan S. (2021). Nesfatin-1 stimulates the hypothalamus-pituitary-interrenal axis hormones in goldfish. Am. J. Physiol. – Regul. Integr. Comp. Physiol..

[20] Ramanjaneya M., Tan B.K., Rucinski M., Kawan M., Hu J., Kaur J., Patel V.H., Malendowicz L.K., Komarowska H., Lehnert H. (2015). Nesfatin-1 inhibits proliferation and enhances apoptosis of human adrenocortical H295R cells. J. Endocrinol..

[21] Özdemir Petek M. (2024). Vardiyalı Çalışan Sağlık Personelinin Serum Nesfatin-1 Düzeyi ile Sirkadiyen Ritim, Enerji Gereksinimi ve Beslenme Durumu İlişkisinin Değerlendirilmesi. Doctoral Dissertation.

[22] Vas S., Ádori C., Könczöl K., Kátai Z., Pap D., Papp R.S., Bagdy G., Palkovits M., Tóth Z.E. (2013). Nesfatin-1/NUCB2 as a Potential New Element of Sleep Regulation in Rats. PLoS ONE.

[23] Price C.J., Samson W.K., Ferguson A.V. (2008). Nesfatin-1 inhibits NPY neurons in the arcuate nucleus. Brain Res..

[24] Dong J., Guan H.-Z., Jiang Z.-Y., Chen X. (2014). Nesfatin-1 influences the excitability of glucosensing neurons in the dorsal vagal complex and inhibits food intake. PLoS ONE.

[25] Toru Ü., Ayada C., Genç O., Akcılar R., Şahin S., Erken G., Erken H.A., Turgut G., Turgut S. (2015). Possible activation of the immune system by chronic peripheral nesfatin-1 application at the acute phase of ischemia/reperfusion injury. Erciyes Med. J..

[26] Szklarczyk D., Kirsch R., Koutrouli M., Nastou K., Mehryary F., Hachilif R., Gable A.L., Fang T., Doncheva N.T., Pyysalo S. (2023). The STRING database in 2023: Protein–protein association networks and functional enrichment analyses for any sequenced genome of interest. Nucleic Acids Res..

[27] Schalla M.A., Stengel A. (2018). Current Understanding of the Role of Nesfatin-1. J. Endocr. Soc..

[28] Schalla M.A., Unniappan S., Lambrecht N.W.G., Mori M., Taché Y., Stengel A. (2020). NUCB2/nesfatin-1—Inhibitory effects on food intake, body weight and metabolism. Peptides.

[29] Zheng J., Han J., Wang Y., Tian Z. (2023). Role of brain NUCB2/nesfatin-1 in stress and stress-related gastrointestinal disorders. Peptides.

[30] Takagi K., Miki Y., Tanaka S., Hashimoto C., Watanabe M., Sasano H., Ito K., Suzuki T. (2016). Nucleobindin 2 (NUCB2) in human endometrial carcinoma: A potent prognostic factor associated with cell proliferation and migration. Endocr. J..

[31] Ren L., Bao D., Wang L., Xu Q., Xu Y., Shi Z. (2022). Nucleobindin-2/nesfatin-1 enhances the cell proliferation, migration, invasion and epithelial-mesenchymal transition in gastric carcinoma. J. Cell. Mol. Med..

[32] Ning S., Liu C., Wang K., Cai Y., Ning Z., Li M., Zeng L. (2023). NUCB2/Nesfatin-1 drives breast cancer metastasis through the up-regulation of cholesterol synthesis via the mTORC1 pathway. J. Transl. Med..

[33] Kmiecik A.M., Dzięgiel P., Podhorska-Okołów M. (2021). Nucleobindin-2/Nesfatin-1-A New Cancer Related Molecule?. Int. J. Mol. Sci..

